# A New Hierarchy of Phylogenetic Models Consistent with Heterogeneous Substitution Rates

**DOI:** 10.1093/sysbio/syv021

**Published:** 2015-04-08

**Authors:** Michael D. Woodhams, Jesús Fernández-Sánchez, Jeremy G. Sumner

**Affiliations:** ^1^School of Physical Sciences, University of Tasmania, Hobart, TAS 7005, Australia and ^2^Departament de Matemàtica Aplicada I, Universitat Politècnica de Catalunya, Barcelona, Spain

**Keywords:** Lie Markov models, Model selection, ModelTest, multiplicative closure, phylogenetics

## Abstract

When the process underlying DNA substitutions varies across evolutionary history, some standard Markov models underlying phylogenetic methods are mathematically inconsistent. The most prominent example is the general time-reversible model (GTR) together with some, but not all, of its submodels. To rectify this deficiency, nonhomogeneous Lie Markov models have been identified as the class of models that are consistent in the face of a changing process of DNA substitutions regardless of taxon sampling. Some well-known models in popular use are within this class, but are either overly simplistic (e.g., the Kimura two-parameter model) or overly complex (the general Markov model). On a diverse set of biological data sets, we test a hierarchy of Lie Markov models spanning the full range of parameter richness. Compared against the benchmark of the ever-popular GTR model, we find that as a whole the Lie Markov models perform well, with the best performing models having 8–10 parameters and the ability to recognize the distinction between purines and pyrimidines.

Exclusively from a mathematical point of view, Sumner et al. (2012a) introduced the Lie Markov models of DNA evolution that have the property of closure under matrix multiplication. We will give a detailed explanation of what is meant by closure and why it is of practical importance, but essentially it ensures that a nonhomogeneous process (where rate matrices change with time while staying within a given model) is equivalent to an “average” homogeneous process using rate matrices obtainable from the same model. Models which do not have this property (notably including general time-reversible model, GTR) have a consistency problem when modeling a nonhomogeneous process (Sumner et al. 2012a): if a sequence evolves for a time under one set of GTR rate parameters, then for a time under a different set of GTR rate parameters, the joint probabilities (pattern frequencies) between the start and end of this process cannot (in general) be described by a single GTR model. One consequence of this is that, in a nonhomogeneous GTR model (i.e., different GTR rate matrices on each branch of a tree), pruning the tree changes the distribution of site patterns achievable at the remaining taxa.

Nonhomogeneous Lie Markov models on a tree will provide consistent estimation, in the presence of a nonhomogeneous model, regardless of taxon sampling. However, in the likelihood testing section of this article we only apply homogeneous Lie Markov models (a single rate matrix across the tree). This is a test to establish the biological plausibility (or otherwise) of each Lie Markov model. To realize the consistency advantages of Lie Markov models requires modeling nonhomogeneous evolution which is a difficult but not insurmountable problem, for example, Jayaswal et al. (2014). We intend to take this step in a future article.

From a mathematician's viewpoint, a “closed” model is defined by the set of Markov matrices in the model being closed under matrix multiplication. From a phylogeneticist's point of view, the property we care about is that we can add or remove taxa without affecting the site patterns that the model can generate over the remaining taxa. We can view this as a closure property on tree pruning, and the phylogeneticist's closure property is implied by the mathematician's closure property. The practical significance of model misspecification that can occur when implementing a model that is not closed under matrix multiplication has been explored by Sumner et al. (2012b).

Sumner et al. (2012a) derived the hierarchy of Lie Markov models with maximal symmetry (those that treat all nucleotides equivalently). This hierarchy consists of the Jukes–Cantor (JC; one-parameter) model (Jukes and Cantor 1969), the K3ST (three-parameter) model (Kimura 1981), the F81 (four-parameter) model (Felsenstein 1981), the general Markov (twelve-parameter) model (Barry and Hartigan 1987), and a previously unknown six-parameter model “F81 + K3ST,” which has rate matrices that are the sum of F81 and K3ST rate matrices. In Sumner et al. (2012b; Table 2), these models were compared to GTR under an Akaike information criterion (Akaike 1974) framework. There it was found that F81 + K3ST was marginally superior to GTR on one data set (human mitochondrial genomes), and markedly inferior on the other four data sets examined. Despite its novelty, a practical disadvantage of the F81 + K3ST model is that it does not account for the biological fact that transitions occur at higher rate than transversions (Kimura 1980, 1981).

It is the purpose of this article to explore a larger hierarchy of “RY” Lie Markov models sensitive to the grouping of nucleotides into purines (R) and pyrimidines (Y). This hierarchy was derived in Fernández-Sánchez et al. (2014) and totals 37 models capable of distinguishing transitions from transversions. Here we present the models in a more accessible way; present some new results on model nesting, equilibrium frequencies, and parameterization; and test the models.

We will start with an example model, RY5.6b, to illustrate the various technical issues that arise when using these models. Next we describe the construction of the Lie Markov models in a manner that is friendly to nonmathematicians, and discuss how the hierarchy of purine/pyrimidine models can be extended to include models distinguishing different DNA pairs. We test the Lie Markov models for biological plausibility on a range of real data sets comparing directly to commonly used time reversible models. We finish with a few technical issues, covering model-nesting relationships, parameterization of the models, and embeddability.

## An Example Lie Markov Model: RY5.6B

To motivate the rest of our discussion, we start by presenting one of the RY Lie Markov models in detail. First, we note some notational conventions and definitions. The column of a Markov matrix (also known as a stochastic, probability, or substitution matrix) or of a rate matrix indicates the initial state of the base, and the row the final state, hence rate matrices have columns which sum to zero (and Markov matrix columns sum to one). Note that this varies from the commonly used rows sum to zero convention. The rows and columns are indexed by the DNA bases in the order A, G, C, T. This deviation from standard alphabetical order groups the purines and pyrimidines, making the relations among matrix entries more apparent. The term “stochastic” when applied to a rate matrix means that all off-diagonal entries are non-negative, and when applied to a Markov matrix means all entries are non-negative. We refer to the number of independent parameters in a Lie Markov model as its “dimension.”

The rate matrices of model RY5.6b can be expressed as

(1) $$Q_{5.6b}=\left(\begin{array}{cccc} -3a+d+e_{1} & a+2a_{2}+d+e_{1} & a-a_{2}+d+e_{1} & a-a_{2}+d+e_{1}\\ a+2a_{2}+d-e_{1} & -3a+d-e_{1} & a-a_{2}+d-e_{1} & a-a_{2}+d-e_{1}\\ a-a_{2}-d+e_{2} & a-a_{2}-d+e_{2} & -3a-d+e_{2} & a+2a_{2}-d+e_{2}\\ a-a_{2}-d-e_{2} & a-a_{2}-d-e_{2} & a+2a_{2}-d-e_{2} & -3a-d-e_{2} \end{array}\right).$$

The “5” in the model name indicates that this is a five-dimensional model, with parameters $a,a_{2},d,e_{1},e_{2}$. (The choice of parameter labels will be explained in the next section.)

The model is five dimensional in the sense that we require five degrees of freedom to specify any rate matrix within the model. Note that we can multiply $Q_{5.6b}$ by a scalar and remain in the model. It is common when considering DNA mutation models to fix the scale of the rate matrix in some manner, otherwise the scale of the rate matrix and the overall scale of tree branch lengths form a redundant pair of parameters. Our preferred manner of fixing the scale is to constrain the rate matrix to have a trace of −4. If the scale is fixed (by whatever method), then this becomes a four-dimensional model.

Note that the entries of the rate matrix are linear expressions in the parameters. This is a feature of all Lie Markov models, but not of the GTR and related models.

The reader should be alarmed by the appearance of minus signs in the off-diagonal entries of the rate matrix in equation (1). Unfortunately, there are no simple constraints on the parameters $a,a_{2},d,e_{1},e_{2}$ which restrict to exactly the set of stochastic matrices of this form. A reformulation solves this problem and illuminates the model structure significantly:

(2) $$Q_{5.6b}=\left(\begin{array}{cccc} {}* & \alpha +\rho_{A} & \beta +\rho_{A} & \beta +\rho_{A}\\ \alpha +\rho_{G} & * & \beta +\rho_{G} & \beta +\rho_{G}\\ \beta +\rho_{C} & \beta +\rho_{C} & * & \alpha +\rho_{C}\\ \beta +\rho_{T} & \beta +\rho_{T} & \alpha +\rho_{T} & * \end{array}\right)$$

where the “*” stands for the values required for the columns to sum to zero. Now Q is stochastic so long as the parameters are all nonnegative: $\alpha ,\beta ,\rho_{A},\rho_{G},\rho_{C},\rho_{T}\geq 0$, but the cost of this reformulation is that we are now using six parameters to express a five-dimensional model. The resulting parameter redundancy is expressed by

$$\begin{array}{l} Q_{5.6b}(\alpha ,\beta ,\rho_{A},\rho_{G},\rho_{C},\rho_{T})=\\ Q_{5.6b}(\alpha +\delta ,\beta +\delta ,\rho_{A}-\delta ,\rho_{G}-\delta ,\rho_{C}-\delta ,\rho_{T}-\delta ), \end{array}$$

for all choices δ. The ability to express the model with six nonnegative parameters is due to the set of stochastic rate matrices of this model forming a “polyhedral cone” having six “rays,” this being the origin of the “6” in the model name. Rays and polyhedral cones in this context are more fully explained in Fernández-Sánchez et al. (2014).

While all the Lie Markov models can be formulated in this way, most of them acquire redundant parameters—in some cases *many* redundant parameters—to ensure stochastic rate matrices. Later in this article we will explore some alternative parameterizations which generate the set of stochastic rate matrices of a Lie Markov model with simple parameter constraints and without redundant parameters.

The matrix (2) also reveals that model 5.6b can be thought of as the sum of the Kimura two-substitution-type (K2ST) model (Kimura 1980) (parameters α, β) and the F81 model (Felsenstein 1981) (parameters $\rho_{A}$, $\rho_{G}$, $\rho_{C}$, $\rho_{T}$). If we changed the additions in matrix (2) to multiplications, we would have the HKY model (Hasegawa et al. 1985). Most of the Lie Markov models are not so easily related to existing models.

The defining features of the RY Lie Markov models (illustrated here by RY5.6b) are 2- fold: First, the Markov matrices obtained from this model are closed under matrix multiplication (this is what makes the model “Lie Markov”). This means that if $M_{1}$ and $M_{2}$ are Markov matrices obtained by taking the matrix exponential of two (distinct) rate matrices from the model, then the product $M_{1}M_{2}$ is obtainable as the matrix exponential of a third RY5.6b rate matrix. Second, the model recognizes the groupings of nucleotides into purines and pyrimidines (this is easily seen by inspection of matrix (2)). The simple idea is that any interchange of nucleotides that preserves the purine/pyrimidine grouping will correspond to a row and column permutation of an RY5.6b rate matrix that will produce another RY5.6b rate matrix.

It is also worth noting that model RY5.6b can have any equilibrium frequencies of bases (under a suitable choice of rate parameters). The easiest way to see this is to notice model 5.6b has F81 as a submodel (i.e., F81 is a special case of RY5.6b), and F81 can have any equilibrium base frequencies (EBF). This is not a general property of Lie Markov models — as noted above, the JC and K3ST models are Lie Markov models, but these have uniform base frequencies at equilibrium. The EBF of the various Lie Markov models are derived below.

## Composition of the Lie Markov Models

Under a continuous-time formulation with time parameter t, a Markov matrix M, whose elements are the probabilities of nucleotide substitutions, is constructed from a rate matrix Q by matrix exponentiation:

$$M=\exp(Qt)=I+Qt+\frac{Q^{2}t^{2}}{2!}+\frac{Q^{3}t^{3}}{3!}+\ldots$$

Fix a model (e.g., GTR or Kimura's K2ST), and take any two rate matrices $Q_{1}$ and $Q_{2}$ from the model. Suppose there exists stochastic $Q^{\prime }$ such that $\exp(Q^{\prime }(t_{1}+t_{2}))=\exp(Q_{1}t_{1})\exp(Q_{2}t_{2})$, we would like $Q^{\prime }$ to be in the same model. Putting aside the caveat “if $Q^{\prime }$ exists”—in most cases $Q^{\prime }$ will exist as long as $Q_{1}$ and $Q_{2}$ are not too different—this would appear a natural condition to ask of a model, especially if one expects some time non homogeneity in the DNA substitution process.

For this property to hold for a given Markov model, Sumner et al. (2012a) have shown that is a sufficient condition that the subset of rate matrices that define the model be:

(i) closed under addition and scalar multiplication (i.e. the set forms a vector space), and
(ii) closed under matrix commutator (Lie) brackets, that is, $\left[Q_{1},Q_{2}\right]:=Q_{1}Q_{2}-Q_{2}Q_{1}$ is also in the space.

For the purpose of these conditions we are forced to include nonstochastic rate matrices in the discussion, for example, $\left[Q_{1},Q_{2}\right]$ is often not stochastic. Together these conditions demand that the model forms a *Lie algebra*. Any continuous time Markov model which satisfies these conditions is referred to as a “Lie Markov model.”

As stated in the introduction, Sumner et al. (2012a) derived the set of Lie Markov models that treat each nucleotide on an equal footing. Fernández-Sánchez et al. (2014) went further and characterized the “RY” Lie Markov models which have a symmetry condition that allow one pairing of DNA bases (canonically the RY pairing: AG and CT) to be treated differently from other pairings. We reiterate the essential results here without further discussion as to how they were obtained.

Each RY Lie Markov model has rate matrices which are a linear combination of basis matrices chosen from a set of 12 (Table 1). Not all subsets of these basis matrices yield a Lie Markov model. The list of the 37 that do is given in Table 2. We adopt a convention that the variable used for the weight of a basis matrix is the same as the basis matrix name, but in lowercase, for example, $e_{1}$ is the weight of $E_{1}$, hence the choice of variable names in equation (1).

**Table 1.** The rate matrices of RY Lie Markov models are linear combinations of basis matrices

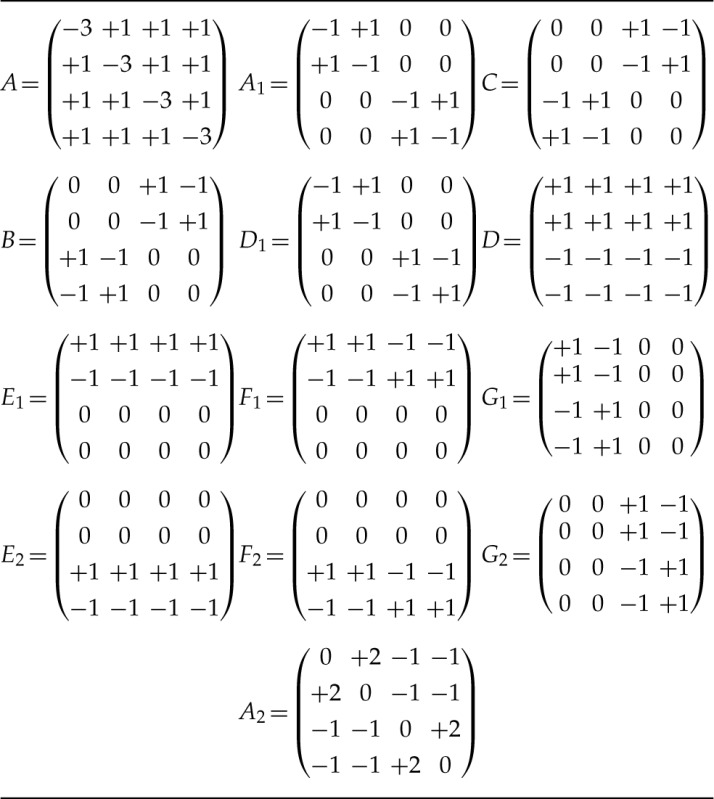

Notes: Each model uses a subset of the first 12 matrices listed here. Under some circumstances it is mathematically convenient to replace $A_{1}$ with the 13th matrix, $A_{2}=3A_{1}-A$.

**Table 2.** The RY Lie Markov models. Basis matrix $A_{2}$ can be substituted for $A_{1}$ throughout

Name	Basis matrices	Name	Basis matrices
1.1	A	6.6	$A,A_{1},B,C,D,D_{1}$
2.2b	$A,A_{1}$	6.7a	$A,A_{1},B,D,E_{1},E_{2}$
3.3a	$A,A_{1},B$	6.7b	$A,A_{1},C,D,E_{1},E_{2}$
3.3b	$A,A_{1},C$	6.8a	$A,A_{1},D,D_{1},E_{1},E_{2}$
3.3c	$A,A_{1},D_{1}$	6.8b	$A,A_{1},D,D_{1},G_{1},G_{2}$
3.4	$A,A_{1},D$	6.17a	$A,A_{1},B,D,G_{1},G_{2}$
4.4a	$A,D,E_{1},E_{2}$	6.17b	$A,A_{1},C,D,G_{1},G_{2}$
4.4b	$A,A_{1},D,D_{1}$	8.8	$A,A_{1},D,D_{1},E_{1},E_{2},F_{1},F_{2}$
4.5a	$A,A_{1},B,D$	8.10a	$A,A_{1},B,C,D,D_{1},E_{1},E_{2}$
4.5b	$A,A_{1},C,D$	8.10b	$A,A_{1},B,C,D,D_{1},G_{1},G_{2}$
5.6a	$A,A_{1},B,C,D_{1}$	8.16	$A,A_{1},D,D_{1},E_{1},E_{2},G_{1},G_{2}$
5.6b	$A,A_{1},D,E_{1},E_{2}$	8.17	$A,A_{1},B,D,E_{1},E_{2},G_{1},G_{2}$
5.7a	$A,A_{1},B,E_{1},E_{2}$	8.18	$A,A_{1},B,D,E_{1},E_{2},F_{1},F_{2}$
5.7b	$A,A_{1},B,F_{1},F_{2}$	9.20a	$A,A_{1},B,C,D_{1},E_{1},E_{2},F_{1},F_{2}$
5.7c	$A,A_{1},B,G_{1},G_{2}$	9.20b	$A,A_{1},B,C,D_{1},F_{1},F_{2},G_{1},G_{2}$
5.11a	$A,A_{1},D_{1},E_{1},E_{2}$	10.12	$A,A_{1},B,C,D,D_{1},E_{1},E_{2},F_{1},F_{2}$
5.11b	$A,A_{1},D_{1},F_{1},F_{2}$	10.34	$A,A_{1},B,C,D,D_{1},E_{1},E_{2},G_{1},G_{2}$
5.11c	$A,A_{1},D_{1},G_{1},G_{2}$	12.12	$A,A_{1},B,C,D,D_{1},$
5.16	$A,A_{1},D,G_{1}G_{2}$		$E_{1},E_{2},F_{1},F_{2},G_{1},G_{2}$

Notes: The number before the point indicates the dimension (number of parameters) of the model, the number after the point is the number of rays generated by the model.

If we take the basis matrices in Table 1 as having rows and columns labeled in our canonical order A, G, C, T, then AG and CT are the distinguished pairings, and we describe this as an RY model. The ordering of bases is immaterial so long as it pairs the purines and pyrimidines. Taking the matrices in Table 1 to be ordered (for example) T, C, A, G as used by PAML (Yang 1997) will yield the same models, just with some permutation and sign changes of weights of basis matrices. Alternatively, if we label the basis matrices in the order A, T, C, G, we distinguish the Watson–Crick pairs AT and CG, which we describe as a WS (Weak/Strong) model. Finally if we label the basis matrices in order A, C, G, T, we distinguish AC and GT and call these MK (aMino,Keto) models. (R, Y, W, S, M, and K are the standard IUPAC ambiguity codes for these pairings.) This allows us to distinguish RY5.6b as model 5.6b with the RY grouping, whereas models WS5.6b or MK5.6b have the same structure but distinguish AT and CG (i.e., the matrix in equation (1) with row/column ordering A,T,C,G), or AC and GT (i.e., the matrix in equation (1) with row/column ordering A,C,G,T), respectively.

If we make statements about (for example) the 5.6b model without “RY,” “WS” or “MK” prefix, the statement applies equally to RY5.6b, WS5.6b, and MK5.6b. Additionally, some of the models have full symmetry, meaning there is no distinction between the RY, WS and MK variants. These are models 1.1 (JC), 3.3a (K3ST), 4.4a (F81), 6.7a (F81 + K3ST), 9.20b (doubly stochastic), and 12.12 (general Markov). These models never get a two-letter prefix. Since there are 37 models listed in table 2, 31 of which have distinct RY, WS, and MK variants, we have 99 models in total. By comparison, the original ModelTest program (Posada and Crandall 1998) compares 14 models and jModelTest2 (Darriba et al. 2012) compares up to 406 models. (These counts are before considering rate variation across sites.)

**Figure 1.**
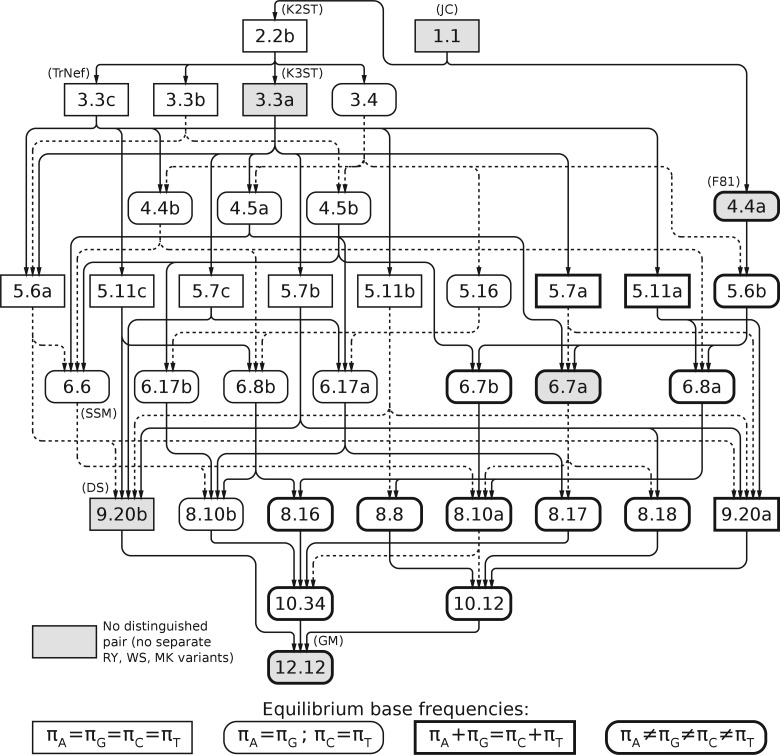
Nesting relationships of the RY Lie Markov models. Box shape and weight indicates the degrees of freedom in EBF. Alternate model names are in parentheses. Solid or dotted connecting lines are to reduce visual confusion and have no additional significance.

A number of these models have already been studied: 1.1 is the JC model (Jukes and Cantor 1969), RY2.2b and 3.3a are the Kimura two- and three-substitution-type models (Kimura 1980, 1981) (also known as the K2ST/K2P/K80 and K3ST/K3P/K81 models), RY3.3c is the Tamura Nei model with equal base frequencies (Tamura and Nei 1993), 4.4a is the F81 model (Felsenstein 1981), WS6.6 is the strand symmetric model (Yap and Pachter 2004; Casanellas and Sullivant 2005), 9.20b is the doubly stochastic model and 12.12 is the general Markov model (Barry and Hartigan 1987). Table 3 lists these model aliases, along with information on time reversibility and EBF which we shall develop later in this article. Some of this information is also reiterated in Figure 1.

**Table 3.** Some properties of the RY Lie Markov models

Name	aka	Rev?	EBFDF	Name	aka	Rev?	EBFDF
1.1	JC	✓	0	6.6	(SSM)	×	1
2.2b	K2ST	✓	0	6.7a		×	3
3.3a	K3ST	✓	0	6.7b		×	3
3.3b		×	0	6.8a		×	3
3.3c	TrNef	✓	0	6.8b		×	1
3.4		✓	1	6.17a		×	1
4.4a	F81	✓	3	6.17b		×	1
4.4b		✓	1	8.8		×	3
4.5a		×	1	8.10a		×	3
4.5b		×	1	8.10b		×	1
5.6a		×	0	8.16		×	3
5.6b		×	3	8.17		×	3
5.7a		×	2	8.18		×	3
5.7b		×	0	9.20a		×	2
5.7c		×	0	9.20b	DS	×	0
5.11a		×	2	10.12		×	3
5.11b		×	0	10.34		×	3
5.11c		×	0	12.12	GM	×	3
5.16		×	1				

Notes: The “aka” (“also known as”) column identifies models already known to phylogenetics under a different name (see text). “Rev?” indicates which models are time reversible (✓) and which are not (×). “EBFDF” is the equilibrium base frequency degrees of freedom. EBFDF = 0 has $\pi_{A}=\pi_{G}=\pi_{C}=\pi_{T}$. EBFDF = 1 has $\pi_{A}=\pi_{G};\pi_{C}=\pi_{T}$. EBFDF=2 has $\pi_{A}+\pi_{G}=\frac{1}{2}=\pi_{C}+\pi_{T}$. EBFDF = 3 has unconstrained EBF.

For the purpose of easy comparison to the presentation given in Fernández-Sánchez et al. (2014), note that we have renamed the basis matrices and added $A_{2}$ as an alternative to $A_{1}$. A table of the basis matrix renaming is in the Supplementary Material available on Dryad at http://dx.doi.org/10.5061/dryad.461g6. We have also omitted model 2.2a, which is of no phylogenetic interest as it forbids transversions entirely. Model 2.2a has basis matrices $A_{1},D_{1}$.

## Likelihood Testing on Real Data

We proceed to investigate how well these models fit real data. We have taken seven diverse aligned DNA data sets and calculated the maximum likelihood under each model. The data sets were chosen to cover a range of DNA types (nuclear, mitochondrial, and chloroplast) and phylogenetic ranges (within a single species to covering a class.)

The data sets are 53 individuals × 16589 sites human mitochondria (of which only 202 sites are variable) (Ingman et al. 2000), 15×89436 (taxa × sites) angiosperm (+ outgroup) chloroplast (Goremykin et al. 2005), 33×1141 cormorants and shags (family Phalacrocoracinae), mixed mitochondria and nuclear (Holland et al. 2010), 8×127026 *Saccharomyces* (+ outgroup) yeast mostly nuclear plus some mitochondria (Rokas et al. 2003), 11×2178 teleost fish nuclear (Zakon et al. 2006), 14×4135 buttercup (genus *Ranunculus*) chloroplast (Joly et al. 2009), and 27×7324 Ratite (bird-order) mitochondria (Phillips et al. 2010).

The models tested are the 99 Lie Markov models discussed above (6 fully symmetric, 31 with RY, WS, and MK variants) and, for comparison, the time-reversible models of the original ModelTest program (Posada and Crandall 1998). ModelTest uses 14 models, but five of these are also RY Lie Markov models (JC = 1.1, K80 = RY2.2b, K81 = 3.3a, TrNef = 3.3c, F81 = 4.4a) so this adds nine models for a total of 108.

Our analysis imitates the procedure used by ModelTest (Posada and Crandall 1998): (i) A neighbor joining tree is created using the JC distances; (ii) The tree is then midpoint rooted (as most RY Lie Markov models are not time reversible, root location is relevant. We midpoint root for simplicity, as we are only establishing model plausibility rather than attempting to construct an accurate phylogeny); (iii) For each model, we find the maximum likelihood by optimizing model parameters and branch lengths (but not tree topology) using a hill-climbing algorithm (the base distribution at the root is assumed equal to the equilibrium distribution of the model); (iv) The optimization is performed for four different models of rate variations across site: single rate, invariant sites (+I), gamma rate distribution (+ Γ, with 8 rate classes), and both invariant sites and gamma distribution (+ I + Γ); and (v) Finally, we apply the Bayesian Information Criterion (BIC) (Schwarz 1978) correction to penalize models with more parameters (Table 4).

**Table 4.** The top 10 models for each data set, by Bayesian Information Criterion (BIC)

Clade:	Human	Angiosperms	Cormorants	Yeast	Teleost Fish	Buttercups	Ratites
Approx range:	Species	Class	Family	Genus	mult. orders	Genus	Order
Tree diameter:	0.008	0.434	0.721	1.465	0.523	0.021	1.085
DNA type	mitoch	chlorop	mito/nuc	mostly nuc	nuclear	chlorop	mitoch
taxa×sites	53×16589	15×89436	33×1141	8×127026	11×2178	14×4135	27×7324
Site rate model	+Γ+I	+Γ	+Γ+I	+Γ+I	+Γ	+I	+Γ+I
$1^{\text{st}}$	TrN	MK10.34	HKY	12.12	RY5.11b	WS4.4b	RY8.16
$2^{\text{nd}}$	HKY	RY8.18	TrN	GTR	RY3.3c	WS3.4	RY10.34
ΔBIC	8.9	16.0	6.5	79.8	0.1	0.0	7.8
$3^{\text{rd}}$	TIM	12.12	K81uf	RY10.12	RY2.2b	WS4.5a	TVM
ΔBIC	9.7	16.9	6.8	912.0	3.4	5.1	8.6
$4^{\text{th}}$	RY8.8	MK8.17	RY8.8	RY8.8	RY5.7b	WS4.5b	12.12
ΔBIC	13.5	26.9	10.8	946.5	6.4	6.0	14.0
$5^{\text{th}}$	RY8.18	WS8.10a	TIM	RY9.20a	TIMef	MK5.7a	GTR
ΔBIC	15.4	27.3	13.3	1156.6	6.7	10.3	15.2
$6^{\text{th}}$	K81uf	WS10.12	RY8.18	WS10.12	RY4.4b	RY5.7a	WS10.12
ΔBIC	18.6	28.3	16.2	1450.6	7.5	10.7	18.6
$7^{\text{th}}$	GTR	RY10.12	MK8.10a	TVM	RY3.4	WS6.8a	RY8.17
ΔBIC	21.3	29.8	17.3	1518.7	8.5	11.5	30.5
$8^{\text{th}}$	TVM	WS10.34	TVM	TIM	SYM	WS5.6b	WS8.10a
ΔBIC	29.9	36.1	19.3	1613.4	9.5	12.4	34.7
$9^{\text{th}}$	RY10.12	RY9.20a	RY10.12	TrN	RY5.11a	WS6.6	MK10.12
ΔBIC	30.5	89.5	20.1	1640.5	9.6	13.1	42.3
$10^{\text{th}}$	MK10.34	WS8.10b	WS8.17	MK10.34	3.3a	K81uf	WS10.34
ΔBIC	31.1	108.5	21.6	1663.0	10.0	14.4	44.6

Notes: ΔBIC is how much worse this model scores than the optimal model (first). A complete table of BIC scores is available in the Supplementary Material available on Dryad at http://dx.doi.org/10.5061/dryad.461g6. Tree diameter is approximately the number of mutations per site between the most distant taxa.

In Table 4, we present BIC scores for the best models for each data set under the optimal rate variation across sites model. (Scores for nonoptimal rate variation models are in the Supplementary Material available on Dryad at http://dx.doi.org/10.5061/dryad.461g6.) For each data set, the models were ranked by BIC and, for each model, these rankings are summarized in Table 5.

**Table 5.** Summary of rankings of models under BIC for the seven data sets. Models marked “*” are time reversible, non-Lie Markov models

Model	Median	Best	EBF	Model	Median	Best	EBF	Model	Median	Best	EBF
	rank	rank	DF		rank	rank	DF		rank	rank	DF
*TVM	8	3	3	WS9.20a	38	28	2	WS8.8	73	22	3
RY10.12	9	3	3	*SYM	41	8	0	MK6.7b	74	42	3
MK10.34	11	1	3	9.20b	41	39	0	WS8.16	75	23	3
WS10.34	11	8	3	RY6.8b	42	34	1	WS6.7b	77	14	3
*GTR	12	2	3	WS6.6	43	9	1	MK8.8	77	39	3
12.12	12	1	3	MK8.10b	43	17	1	WS6.8a	78	7	3
RY8.18	14	2	3	RY8.10b	46	33	1	WS5.6b	79	8	3
RY8.8	14	4	3	WS8.10b	47	10	1	MK6.8a	79	40	3
*K81uf	15	3	3	RY6.6	48	33	1	MK8.16	81	38	3
*TIM	16	3	3	*TVMef	49	13	0	WS5.11a	81	50	2
WS8.10a	17	5	3	RY4.4b	49	6	1	WS6.17b	82	16	1
MK8.17	17	4	3	MK6.6	50	18	1	MK5.6b	83	34	3
*HKY	18	1	3	RY5.11b	50	1	0	WS6.8b	87	12	1
*TrN	18	1	3	RY5.11c	50	15	0	WS5.16	87	11	1
WS10.12	18	6	3	RY5.16	51	39	1	MK5.11a	88	37	2
RY9.20a	19	5	2	RY4.5a	53	18	1	MK4.5b	88	69	1
MK10.12	20	9	3	MK6.17a	54	32	1	WS5.11b	88	65	0
RY8.16	21	1	3	RY6.17a	54	34	1	WS4.5b	89	4	1
RY8.10a	23	11	3	MK5.6a	55	19	0	MK4.4b	89	64	1
RY10.34	23	2	3	RY6.17b	57	35	1	WS3.3b	90	59	0
MK8.10a	24	7	3	MK4.5a	58	25	1	WS3.4	91	2	1
RY6.8a	24	13	3	RY5.7b	59	4	0	MK6.8b	91	67	1
6.7a	25	13	3	*TIMef	60	5	0	WS4.4b	92	1	1
MK8.18	25	16	3	RY3.4	60	7	1	WS2.2b	93	57	0
RY8.17	26	7	3	WS5.6a	61	23	0	MK6.17b	94	71	1
RY5.6b	26	21	3	RY5.6a	61	14	0	WS3.3c	95	56	0
WS8.17	27	10	3	RY4.5b	61	22	1	MK5.16	96	70	1
WS8.18	28	24	3	RY5.7c	61	43	0	WS5.11c	97	67	0
RY6.7b	31	22	3	WS5.7c	62	16	0	MK5.11b	97	85	0
RY5.11a	32	9	2	MK5.7b	63	37	0	MK3.4	98	68	1
MK5.7a	32	5	2	MK5.7c	64	17	0	MK3.3c	98	75	0
WS6.17a	32	15	1	WS5.7b	64	40	0	4.4a	99	44	3
RY5.7a	33	6	2	3.3a	65	10	0	MK5.11c	99	78	0
MK9.20a	33	19	2	RY3.3c	67	2	0	MK3.3b	100	77	0
WS5.7a	35	23	2	RY3.3b	71	11	0	MK2.2b	102	74	0
WS4.5a	37	3	1	RY2.2b	72	3	0	*JC	108	83	0

Notes: EBFDF = Equilibrium base frequency degrees of freedom (see text under “Equilibrium base frequencies”). The best ranked models have high EBFDF.

The top-ranked model, TVM (transversional model), is GTR with a constraint that the two transition rates be equal. A trio of 10-dimensional Lie Markov models follow, then GTR and the general Markov model. The time-reversible models compete well, taking about half the top spots despite being fewer in number than the Lie Markov models. The most successful Lie Markov models are all parameter-rich, having eight (same as TVM) or more dimensions. We caution against reading too much into these rankings, due to the small size of the sample. The most compelling point for this article is that at least some of the Lie Markov models are competitive with established models.

We expect the WS and MK models to do poorly since they do not recognize the established biological preference for transitions over transversions. They do indeed dominate the bottom of the table, however the top of the table shows only slight preference for RY over MK or WS models.

Some models score poorly overall, but score well for one data set. The top-ranked models for the fish data set are RY5.11b and RY3.3c (median ranks 50 and 67). The top-ranked models for the buttercup data set are WS4.4b and WS3.4 (median ranks 92 and 91). We will have more to say on the buttercup results later in this section, and the fish data set in the next section.

The corrected AIC (AICc) (Akaike 1974; Hurvich and Tsai 1989) penalizes extra parameters much less than the BIC. An analysis using AICc in place of BIC is given in the Supplementary Material available on Dryad at http://dx.doi.org/10.5061/dryad.461g6. Under AICc ranking, the top four models are 12.12 (general Markov model), RY10.12, RY8.8, RY8.18, and then GTR.

Despite model RY5.6b's structural similarity to HKY (discussed in the section on RY5.6b), it does not perform well in comparison to HKY ranking 26th to HKY's 13th. Model 6.7a (the sum of K3ST and F81) ranks better (23rd), but still well below HKY.

Model RY8.8 performed well (especially under AICc) and is of particular interest since it is the smallest Lie Markov model that contains all Markov matrices obtainable by multiplying different HKY Markov matrices (the curious reader will be interested to learn the corresponding closure of GTR is the General Markov model, 12.12). For reference, the RY8.8 rate matrix can be parameterized as

$$Q_{8.8}=\left(\begin{array}{cccc} {}* & a & e & e\\ b & * & f & f\\ g & g & * & c\\ h & h & d & * \end{array}\right).$$

The buttercup data set produced results markedly different from the rest, highly ranking WS models with few parameters, and ranking RY models poorly in general. The top four models are all submodels of WS6.6, the strand symmetric model (Casanellas and Sullivant 2005). It appears that the assumptions behind the strand symmetric model, and the WS models generally, hold for these chloroplast sequences (which are largely intergenic spacers (Joly et al. 2009)). This observation is an excellent example of how the nesting relationships of the Lie Markov models can be used to uncover additional information regarding specifics of past evolutionary processes.

In conclusion, we see that for a given data set, we can generally find a Lie Markov model which outscores a time-reversible model, although time-reversible models perform well in comparison to the full set of Lie Markov models. Model RY8.8 stands out as one of the best performing, while also having theoretical justification as the closure of the HKY model. Unexpectedly, models with other base pairings (WS and MK) can also score well for particular models (MK10.34, WS10.34) or particular data sets (buttercups).

## The Structure of RY Models

### Nesting of the Models

When all the rate matrices in model A also occur in model B, we say model A is nested within model B—that is, by adding constraints to model B we can create model A. We may wish to know these relationships so that we can justify using a likelihood-ratio test, or to use the optimal solution for model A as an initial solution for optimizing model B, or for an MCMC analysis which allows switching between related models. The nesting relationships of the RY Lie Markov models can easily be derived from the basis matrix specifications of the models, given in Table 2. The hierarchy of nestings is shown in Figure 1.

Model 6.7a is the F81 + K3ST model. This model has full symmetry, and so is simultaneously in the RY, WS, and MK model families. This means that in some cases, low- parameter models in one-model family are nested within high-parameter models of another, for example RY5.7a, being nested in 6.7a is (by transitivity) also nested in WS8.10a.

A model is “doubly stochastic” if the rows of its rate matrices always sum to zero (in addition to the columns sum to zero condition required of a rate matrix). The most general model with this property is the “doubly stochastic model,” which is model 9.20b in our hierarchy. All models nested within 9.20b also have the doubly stochastic property, for example, 3.3a (K3ST) and 1.1 (JC).

### Equilibrium Base Frequencies

An important property of a model is the range of EBF it can produce. If the base frequencies in the data differ greatly from the EBF of the model, a poor-likelihood score is inevitable. The EBF of a given rate matrix is its principal right eigenvector, which will have eigenvalue zero (as a consequence of the columns-sum-to-zero constraint). The same applies for a Markov matrix, except that the eigenvalue will be one.

The doubly stochastic property implies flat EBF, as $\left(\frac{1}{4},\frac{1}{4},\frac{1}{4},\frac{1}{4}\right)$ is an eigenvector of any doubly stochastic Markov matrix, with eigenvalue one, and hence the EBF for 9.20b has zero degrees of freedom, with EBF $\pi_{A}=\pi_{G}=\pi_{C}=\pi_{T}=\frac{1}{4}$. Nine of the basis matrices (Table 1) have this doubly stochastic property, those nine being A,A1,B,C,D1,F1,F2,G1 and G2, which are also basis matrices of 9.20b, the most general doubly stochastic model. Any model whose basis matrices come from this set will also be doubly stochastic and so have flat EBF. These models (the submodels of 9.20b) are 1.1, 2.2a, 2.2b, 3.3a, 3.3b, 5.6a, 5.7b, 5.7c, 5.11b, and 5.11c.

The remaining three basis matrices are D, $E_{1}$, and $E_{2}$. Each matrix adds one degree of freedom to the EBF distribution. The simplest model to contain all three is 4.4a, the F81 model (Felsenstein 1981). This model has the maximum of three degrees of freedom in its EBF since $\pi_{A}+\pi_{G}+\pi_{C}+\pi_{T}=1$. Supermodels of 4.4a also have full EBF freedom, being 5.6b, 6.7a, 6.7b, 6.8a, 8.8, 8.10a, 8.16, 8.17, 8.18, 10.12, 10.34, and 12.12.

Models which contain D but not $E_{1}$ and $E_{2}$ have $\pi_{A}=\pi_{G}\neq \pi_{C}=\pi_{T}$ (one degree of freedom). (This equation holds for RY models; WS and MK have similar equations.) These models are 3.4, 4.4b, 4.5a, 4.5b, 5.16, 6.6, 6.8b, 6.17a, 6.17b, and 8.10b.

Any model with $E_{1}$ or $E_{2}$ has both, and the models containing these two but not D are 5.7a, 5.11a, and 9.20a. The EBF of these models have two degrees of freedom, $\pi_{A}+\pi_{G}=\pi_{C}+\pi_{T}=\frac{1}{2}$ (for the RY models).

These degrees of freedom are indicated in Figure 1. Table 5 demonstrates that models with many EBF degrees of freedom generally outperform those with few degrees of freedom. We now can understand the unusual choice of models for the fish data set: the top three models (RY5.11b, RY3.3c and RY2.2b) all have zero EBF degrees of freedom. Because this data set is unusual in having close to flat base frequencies (24.4% A, 25.2% G, 23.1% C, 27.4% T), it is able to accept these models where the other data sets strongly reject them.

In contrast to GTR, the relationship between EBF and model parameters for Lie Markov models is often not simple. For example, for model RY5.6b (equation 1) the EBF are:

$$\begin{array}{l} (\pi_{A},\pi_{G},\pi_{C},\pi_{T})=(\frac{1}{4},\frac{1}{4},\frac{1}{4},\frac{1}{4})+\frac{1}{4p}(q+2e_{1},q-2e_{1},\\ -q+2e_{2},-q-2e_{2}) \end{array}$$

where $p=2a+a_{1}$ and $q=2d+\frac{a_{1}d}{a}$. A general formula for the EBF is given in the Supplementary Material available on Dryad at http://dx.doi.org/10.5061/dryad.461g6.

Only a few of the Lie Markov models presented here are time reversible, namely 1.1, 2.2a, 2.2b, 3.3a, 3.3c, 3.4, 4.4a, and 4.4b (Table 3). In the context of a time nonhomogeneous mutation process, we expect base frequencies to be out of equilibrium, so a time-reversible analysis is inappropriate in any case. In this circumstance, there is no advantage to a time-reversible model, so we do not regard the nonreversibility of our models as a major drawback. Time reversibility is a computational convenience, not a law of nature.

In passing, it would be interesting to see how well these restricted EBFs would work with standard time-reversible models. Programs such as jModelTest2 allow only the extremes of zero or three degrees of freedom, but it is plausible that for many data sets three degrees of freedom is overparameterizing, yet zero degrees is insufficient.

## Parameterizations

In the RY5.6b example, we briefly alluded to the problem of generating rate matrices that are stochastic, that is, all off-diagonal elements are nonnegative. We seek parameterizations of the Lie Markov models for which: (1) simple bounds on the parameters (i.e., not dependent on the values of other parameters) restrict the resulting rate matrices to be stochastic; (2) all stochastic rate matrices in the model can be generated from parameters within the bounds; and (3) that slightly different rate matrices can always be specified by slightly different parameters (i.e., the inverse transformation of rate matrix to parameters is continuous).

These conditions allow us to conduct likelihood optimizations by hill climbing. The simple bounds give us a well-defined region of parameter space to search. Condition (2) ensures that all legitimate solutions lie within the space to be searched. Condition (3) ensures the hill climb does not get blocked by a parameterization boundary. We will now derive such a parameterization.

A DNA rate matrix is defined by its 12 off-diagonal elements, so DNA rate matrices lie within a 12-dimensional space. The portion of this space that is stochastic can be equated to the general Markov model, and less general models are subsets of it, generally of lower dimension. The corresponding regions of stochasticity describe a geometric entity known as a convex polydreal cone. The interested reader is referred to Fernández-Sánchez et al. (2014) for details.

In the context of this section, it simplifies matters to take $A_{2}$ as a basis matrix in place of $A_{1}$ (Table 1). Then, all matrices $B_{i}\neq A$ from Table 1 are orthogonal to A, and span the space of rate matrices with trace zero. In particular, the scale (trace) of the rate matrix is determined only by a, the weight of A, and that for fixed a, none of the other weights can go to infinity without violating stochasticity. It follows that the set of rate matrices with a fixed trace defines a bounded set.

For example, model 3.4 has rate matrix:

$$Q_{3.4}=\left(\begin{array}{cccc} -3a+d & a+2a_{2}+d & a-a_{2}+d & a-a_{2}+d\\ a+2a_{2}+d & -3a+d & a-a_{2}+d & a-a_{2}+d\\ a-a_{2}-d & a-a_{2}-d & -3a+d & a+2a_{2}-d\\ a-a_{2}-d & a-a_{2}-d & a+2a_{2}-d & -3a+d \end{array}\right),$$

so the stochasticity constraints can be expressed as:

(3) $$\begin{array}{l} a+2a_{2}+d\geq 0,\\ a+2a_{2}-d\geq 0,\\ a-a_{2}+d\geq 0,\\ a-a_{2}-d\geq 0. \end{array}$$

**Figure 2.**
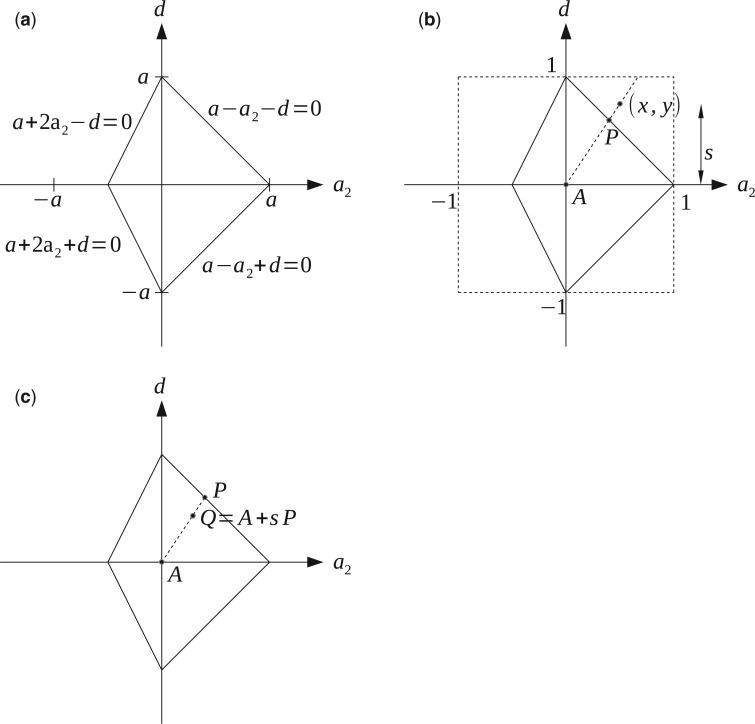
A parameterization of model 3.4 which is restricted to only the stochastic rate matrices. (a) The region of stochasticity for model 3.4 with fixed a. (b) Without loss of generality, we take a=1. Given (x,y) in ${\left[-1,1\right]}^{2}$ defines point (representing a matrix) P on the edge of the region of stochasticity, and s=max(|x|,|y|) a measure of how far (x,y) is from the origin, which defines the JC matrix A. (c) (x,y) have defined a stochastic rate matrix Q(x,y)=A+sP.

This is shown graphically in Figure 2a.

We refer to our preferred parameterization of the RY Lie Markov models as the Cartesian parameterization (it is illustrated for model 3.4 in Fig. 2b,c). From a choice of parameters, this parameterization will produce a stochastic rate matrix Q within the model, and with some given trace. In general, we are given an n dimensional RY Lie Markov model, having basis matrices $A,B_{1},\ldots B_{n-1}$ (where the $B_{i}$ stand for non-A basis matrices from Table 1 as above). Next, we proceed to describe the parameterization in three steps:

1. Generate a matrix
  P′=∑ibiBi, where all bi∈[−1,1].
  The weights $\left(b_{1},\ldots ,b_{n-1}\right)$ are taken as the parameters.
2. Define the “perturbation” matrix P by
  $$P=\frac{1}{-\min(P^{\prime })}P^{\prime }$$
  where $\min(P^{\prime })$ is the minimum off-diagonal element in $P^{\prime }$. Note all the $B_{i}$ have off-diagonal elements summing to zero, therefore $P^{\prime }$ will always contain a negative off-diagonal element (unless it is zero.) Therefore $\min(P^{\prime })<0$ except if $P^{\prime }=0$.
3. Now we find the “saturation” value by
  $$s=\max_{i}|b_{i}|$$
  and finally our rate matrix is
  $$Q=A+sP=A-\frac{s}{\min(P^{\prime })}P^{\prime }$$

If s=1, Q will be on the boundary of stochasticity, having (at least) one off-diagonal element equal to zero, as A has all off-diagonal elements equal to one.

The map from the $b_{i}$ to Q defined as above is one to one, and parameterizes uniformly the section of the stochastic cone with trace −12 taking as parameter space the hypercube ${\left[1,1\right]}^{n-1}$. Should a different fixed scale be desired, we can multiply by a constant. Should we wish the scale of Q to be variable, we can add a scale parameter.

The essence of this method is that the ratios of the $b_{i}$ define the direction in which we will deviate from the JC matrix A, and the overall scale of the $b_{i}$ sets how far we travel from JC toward the boundary of stochasticity. We can also think of it geometrically, as using the $b_{i}$ to form a hypercube enclosing the hyperpolyhedron which is the region of stochasticity, and then shrink-wrapping the hypercube around the hyperpolyhedron. While this parameterization gives Q as a continuous function of the $b_{i}$, it is not a smooth function, and so may not work well with hill-climbing methods which calculate partial derivatives.

We will briefly describe three alternative parameterizations which we explored prior to settling on the Cartesian parameterization described above. Given the stochasticity inequalities (e.g., equations (3) for model 3.4) we can progressively eliminate variables by Fourier–Motzkin elimination (Motzkin 1936). This gives us a parameterization where, having used $x_{1},\ldots ,x_{k}$ to set the weights of $B_{1},\ldots ,B_{k}$, we know the allowable range of weights for $B_{k+1}$ which will keep stochasticity, and we linearly transform $x_{k+1}$ appropriately. The disadvantage of this parameterization is that we need extra computer code specific to each model to implement the Fourier–Motzkin-derived transformation. The *Mathematica* file in the Supplementary Material available on Dryad at http://dx.doi.org/10.5061/dryad.461g6 derives Fourier–Motzkin transformations for each of the models.

The Cartesian parameterization uses the ratios of n−1 parameters to determine a direction and the scale of the parameters to determine a distance. We can separate these roles and use n−2 parameters to specify a direction and supply the “saturation” directly as the $n-1^{\text{th}}$ parameter, that is, we use polar coordinates in the space of matrices with zero trace. In the shrink wrap analogy described above, this corresponds to shrink-wrapping a hypersphere rather than hypercube. The weakness of this method is that the inverse transformation is noncontinuous: Q matrices which are close to each other may not have parameters which are close to each other, due to an angle wrapping from 2π to zero. We tested an extension where angles were unbounded and the radius parameter was in [−1,1] instead of [0,1] (which means the parameter to rate matrix mapping is no longer 1:1.) This helped, but optimization still often failed to find the best likelihood.

Finally, we can form a rate matrix as a sum of nonnegatively weighted ray matrices. While this is simple to code and gives a continuous and smooth function, for most models this uses more parameters than there are dimensions to the model, that is, it overparameterizes, resulting in redundancy and slower optimizations. (For our software, it is slower by about 25% averaged over all models.)

## Embeddability

Multiplicative closure can be tested by taking stochastic rate matrices $Q_{1}$ and $Q_{2}$ from a model and calculating $Q^{\prime }=\log(\exp(Q_{1})\exp(Q_{2}))$ (where “log” is the matrix logarithm). The desired result is that $Q^{\prime }$ be stochastic and in the model.

There are three possible failure modes: (i) the matrix logarithm can produce complex values, so $Q^{\prime }$ may be complex and hence not stochastic; (ii) $Q^{\prime }$ may be real but not stochastic; or (iii) $Q^{\prime }$ may not be in the model. In the Markov chain literature, the property of $Q^{\prime }$ being stochastic is called “embeddability,” and it is discussed at length in the context of phylogenetics and time nonhomogeneous DNA models by Verbyla et al. (2013). General Lie theory tells us that the last of these failure modes should not be a possibility for a Lie Markov model, however we included this possibility in what follows as a sanity check.

We made a preliminary Monte Carlo investigation to get some feeling of how often these failures occur. For each model, we repeatedly generate two random rate matrices $Q_{1}$ and $Q_{2}$ within the model and having predetermined trace, and calculate $Q^{\prime }$. We determine whether this $Q^{\prime }$ is stochastic, real, and in the model. As the traces of $Q_{1}$ and $Q_{2}$ get larger, the chances of nonstochastic (or nonreal) $Q^{\prime }$ grows. In Table 6, we show the level of saturation before about 5% of random products give a nonstochastic (or nonreal) $Q^{\prime }$. (one expected substitution per site corresponds to a trace of −4.) By this measure, the worst performing model was 10.12, which achieved this 5% nonembeddability threshold with trace about −3.3. We observed no instances of $Q^{\prime }$ not being in the model, even when $Q^{\prime }$ is complex. These Monte Carlo calculations are carried out in the *Mathematica* notebook included in the Supplementary Material available on Dryad at http://dx.doi.org/10.5061/dryad.461g6.

**Table 6.** Approximate levels of saturation of model Markov matrices before their product matrix has significant (>5%) chance of being nonembeddable (i.e., “average” rate matrix $Q^{\prime }$, as defined in the text, is nonstochastic)

Saturation	Possible embeddability issues
1 Substitution/site	5.6a, 6.6, 6.8a, 6.8b, 8.8, 8.10a,
	8.10b, 8.16, 8.17, 8.18, 10.12, 10.34
2 Substitution/site	5.6b, 5.7b, 5.11a, 5.11b, 5.11c,
	5.16, 6.7a, 6.7b, 6.17a, 6.17b
3 Substitution/site	4.4b, 5.7c
> 3 Substitution/site	3.4, 4.5a, 4.5b
never	2.2b, 3.3a, 3.3b, 3.3c, 4.4a

Notes: Data derived from Monte Carlo simulation.

The theoretical results of (Sumner et al. 2012a) and (Fernández-Sánchez et al. 2014) prove only that the Lie Markov models have “local multiplicative closure.” This means that the “average” rate matrix of a time varying process can be nonstochastic or even complex. Here, we see that “local” is really quite broad: phylogenies have to be quite deep before nonembeddability potentially becomes an issue, and very deep before the average Q becomes complex. Under most practical circumstances where we would be attempting to reconstruct phylogenies from real data, the Lie Markov models can safely be considered to be simply “multiplicatively closed,” without further reference to the “local” condition.

It is natural to expect that the more different $Q_{1}$ and $Q_{2}$ are, the more likely it is that Q will be nonstochastic. We tested this on models 6.6, 8.8, 8.10b, and 10.12 (see the *Mathematica* notebook). Using a trace value for ($Q_{1}$, $Q_{2}$) which resulted in nonembeddability rate close to 50%, we generated a thousand random $\left(Q_{1},Q_{2}\right)$ pairs, then measured the difference $\left|Q_{1}-Q_{2}\right|$ (where |…| indicates the root mean square of off-diagonal elements). The mean difference for nonembeddable pairs was higher than for embeddable pairs, but only by about 0.3 standard deviations, so embeddability is only weakly dependent on the difference between the input rate matrices.

To compare these results to our likelihood analysis, we found a tree diameter for each data set: we optimized each data set with model 12.12 (general Markov model) with invariable sites (+ I), then the tree diameter is measured as the maximum distance between any two taxa. The results are shown in Table 4. In this analysis, the rate matrices were constrained to have trace −4, which in turn means that the units of branch length are approximately mutations per site.

We see that the data set with the most mutations per site was the yeast data set, having tree diameter of almost 1.5. The worst performing Lie Markov model (from an embeddability point of view) was 10.12, which reached 5% chance of nonembeddability for $Q_{1}$ and $Q_{2}$ having trace −3.3 each, which corresponds to a tree diameter of 1.65. So we see that for the highest mutation rate data set and the most embeddability-sensitive model, the chances of an embeddability problem are below 5%.

The impact of embeddability problems, should they occur, is low. When considering the rate matrix to put on a branch and demanding that the rate matrix be stochastic, we only exclude parts of the parameter space which could be allowed by applying two (or more) distinct stochastic rate matrices over different portions of that branch.

## Discussion

If we model DNA mutation as nonhomogeneous across a phylogeny, using a model which does not have multiplicative closure leads to a lack of consistency (Sumner et al. 2012b). With such a model, applying a single set of model parameters to a given edge cannot reproduce the effects of model parameters varying with time along that edge. The Lie Markov models were developed to avoid this problem (Sumner et al. 2012a). The fully symmetric Lie Markov models are few in number (1.1 (JC), 3.3a (K3ST), 4.4a (F81), 6.7a (K3ST + F81), 9.20b (doubly stochastic), and 12.12 (GM)). By relaxing the symmetry condition to allow one pairing of DNA bases to be distinguished, we greatly increase the number of available models while also allowing for the transition/transversion (RY) distinction which is common in DNA models (e.g. K2ST, HKY). We call the Lie Markov models which allow for the RY distinction as the RY Lie Markov models, although we include within this category the models which distinguish the WS and MK base pairings also.

A classification of the RY Lie Markov models was derived in Fernández-Sánchez et al. (2014), with emphasis on the mathematical derivation and structure of the models. In addition to the fully symmetric Lie Markov models, a further 32 Lie Markov models were found to exist, most of which are novel. In this article we have presented the models in a more accessible way, explored their applicability to real data sets, and dealt with implementation issues around how to parameterize the models. For the 31 useful RY Lie Markov models, we also considered allowing alternative base pairs to be distinguished: the WS pairing and the MK pairing. The WS pairing is more natural to consider than RY for sequences where there is no distinction between the DNA strands, as is usually the case for noncoding DNA.

We compared the performance of the Lie Markov models to the standard benchmark of the GTR model and popular submodels. The majority of Lie Markov models are not time reversible, but we argue that in the context of a nonhomogeneous mutation process, time reversibility has already been lost, so, beyond algorithmic details, this is not a modeling disadvantage.

We tested the models on a diverse set of eukaryotic DNA data sets. For each data set, we fixed the tree topology and then optimized the log-likelihood over model parameters and branch lengths. The optimal log-likelihoods of the models were compared via the BIC. A selection of more traditional time-reversible models were included in the analysis for purposes of comparison. The results show that the RY Lie Markov models are biologically plausible, with five of the seven data sets selecting a Lie Markov model as the optimal model (although in one case, the model is the previously studied General Markov model). One data set (of buttercup chloroplast mostly intergenic DNA) stood out from the rest as strongly favoring WS Lie Markov models with one degree of freedom in base frequencies. This result highlights the usefulness of considering base pairings other than RY, and base frequencies other than uniform or fully unconstrained. This lesson can be extended to time-reversible models also.

We have shown how the basis matrix structure of the RY Lie Markov models determines the nesting relationships of the models, and the EBF that the models can generate. Additionally, when implementing the Lie Markov models, the problem of parameterizing the space of stochastic rate matrices is nontrivial. We have presented a parameteriziation which successfully achieves this, with relative simplicity.

To study the “local” character of the multiplicative closure of Lie Markov models, we performed some Monte Carlo simulations to conclude that multiplicative closure (i.e., a real, stochastic average rate matrix) is very likely to be maintained in all phylogenetic analyses except those with very deep divergences (for which, as sequences are nearly uncorrelated across deep divergence, the choice of model is not very important anyhow).

Most of the Lie Markov models are not time reversible. Modeling a nontime reversible process adds a few complications: the location of the root becomes material (effectively increasing complexity by one taxon), as do the base frequencies at the root, and if we have multiple rate classes, the root base frequencies may be different for the different rate classes. For a nonhomogeneous mutation process, these complications exist in any case, so the non time-reversibility of Lie Markov models costs nothing in that context.

Our future plans include testing the models in a nonhomogeneous context, performing likelihood analysis on many more data sets, and expanding the range of software which implements the models.

## Software

Supplementary tables and figures contain a general formula for equilibrium base frequencies, a colour version of figure 1, the translation between basis matrix names used in Fernández-Sánchez et al. (2014) and this paper, a complete listing of BIC values and ranking of models, a summary of AICc ranking of the models and Fourier–Motzkin parameterizations for each model.

A Mathematica notebook derives the Fourier-Motzkin parameterizations, performs the tests in the embeddability section, and has derivations of nesting, equilibrium base frequencies and time reversibility. We provide source and executable for the Java program used for the likelihood analysis, along with a spreadsheet which collates the results. We caution that the program is experimental and does not have the user interface or robustness suitable for a public release.

The reference implementation of the Lie Markov models is a Beast2 plugin (Bouckaert et al. 2014), available at https://github.com/MichaelWoodhams/BeastLieMarkov.

## Supplementary Material

Data available from the Dryad Digital Repository: http://dx.doi.org/10.5061/dryad.461g6.

## Funding

This work was supported by the Australian Research Council (ARC Future Fellowship FT100100031 and Discovery Early Career Fellowship DE130100423), by the Spanish grant (Ministerio de Economa y Competitividad, MTM2012-38122-C03-01/FEDER) and the Catalan grant (GENCAT 2014SGR 634).
